# A Novel Mechanism of PPAR*γ* Regulation of TGF*β*1: Implication in Cancer Biology

**DOI:** 10.1155/2008/762398

**Published:** 2008-07-03

**Authors:** Chang Ho Lee, Hyung Don Kim, Sang Mi Shin, Sang Geon Kim

**Affiliations:** ^1^Department of Pharmacology, Institute of Biomedical Science, College of Medicine, Hanyang University, Seoul 133-791, South Korea; ^2^Department of Medicine, College of Medicine, Chung-Ang University, Seoul 156-756, South Korea; ^3^Innovative Drug Research Center for Metabolic and Inflammatory Disease, College of Pharmacy, Seoul National University, Seoul 151-742, South Korea

## Abstract

Peroxisome proliferator-activated receptor-*γ* (PPAR*γ*) and retinoic acid X-receptor (RXR) heterodimer, which regulates cell growth and differentiation, represses the TGF*β*1 gene that encodes for the protein involved in cancer biology. This review will introduce the novel mechanism associated with the inhibition of the TGF*β*1 gene by PPAR*γ* activation, which regulates the dephosphorylation of Zf9 transcription factor. Pharmacological manipulation of TGF*β*1 by PPAR*γ* activators can be applied for treating TGF*β*1-induced pathophysiologic disorders such as cancer metastasis and fibrosis. In this article, we will discuss the opposing effects of TGF*β* on tumor growth and metastasis, and address the signaling pathways regulated by PPAR*γ* for tumor progression and suppression.

## 1. INTRODUCTION

Peroxisome proliferator-activated receptor-*γ* (PPAR*γ*) as a ligand-activated transcription factor belongs to the members of nuclear hormone receptor superfamily. PPAR*γ* is implicated in a wide variety of cellular functions, regulating the expression of gene networks required for cell proliferation, differentiation, morphogenesis, and metabolic homeostasis. The transforming growth factor isoforms (TGF*β*1, *β*2, and *β*3) as the members of the TGF*β* superfamily are ubiquitously expressed cytokines [1, 2]. TGF*β* exerts multiple functions with differential expression pattern in organs: each form of TGF*β* has similar biological activities [3]. Among the TGF*β* forms, it is recognized that TGF*β*1 plays a major role in the regulation of cell proliferation and differentiation. In this review paper, we will discuss the role of PPAR*γ* on TGF*β* gene expression.

Accumulating evidences suggest that the interplay of PPAR*γ* and TGF*β* contributes to the regulation of cell proliferation, differentiation, and their associated cellular functions. For instance, the interaction of PPAR*γ* signaling with the proteins affected by the activation of TGF*β* receptor determines the outcome of the breast tumor progression [4]. Many studies have shown that agonist-induced activation of PPAR*γ* interferes with TGF*β*/Smad-dependent or Smad-independent signaling in different cell types [5–12]. The crosstalk between PPAR*γ* and TGF*β* can be achieved not only by PPAR*γ*-dependent modulation of the propagation of TGF*β*/TGF*β* receptor-mediated signaling pathways, but also by the regulation of TGF*β*1 expression itself and TGF*β*1-inducible target genes. Hence, suppression of TGF*β* signaling by PPAR*γ* could be counteracted by the inhibitory action of TGF*β* on the PPAR*γ*-mediated signaling [13–15].

The TGF*β*1 expression is regulated at multiple levels. Diverse transcription factors are involved in the transcriptional regulation of TGF*β* gene expression and post-translational modification makes precursors bound with TGF*β*1 binding proteins mature to TGF*β* molecule [16, 17]. The role of PPAR*γ* activation in TGF*β*1 gene repression has been examined by the experiments using thiazolidinedione PPAR*γ* agonists [18, 19]. These studies on the regulation of the TGF*β*1 gene and the molecular interaction of ligand-activated nuclear receptors for the activation of responsible transcription factor(s) brought insights into the transcriptional control mechanism. The research results showed that PPAR*γ* activation might transrepress the TGF*β* gene, interfering with TGF*β* signaling and thereby altering the expression of TGF*β*-inducible target genes [18], substantiating the fact that ligand activation of PPAR*γ* modulates TGF*β* receptor-mediated gene regulation.

## 2. TGF*β* AND CANCER CELL BIOLOGY

TGF*β*1 exerts its diverse biological effects by acting on distinct combinations of type I and type II receptors and recruiting downstream signal transducers including Smads, consequently regulating a group of target gene expression responsible for a specific biological activity. Smad proteins are classified into R-Smads (receptor-regulated Smads: Smads 1, 2, 3, 5, and 8), Co-Smads (common mediator Smad: Smad 4), and I-Smads (inhibitory Smads: Smad 6 and 7), and these play roles as the transcriptional regulators for the superfamily of TGF*β*1-inducible target genes [1, 2, 20–22]. Smad 2 and Smad 3 are the specific mediators of TGF*β*1, whereas Smad 1, Smad 5, and MADH6/Smad 9 are crucial for bone morphogenic protein signaling [22]. In particular, Smad 3 is involved in the TGF*β*1 gene regulation, which is crucial for the autocrine function of TGF*β*1 [23].

Following the activation of the TGF*β*1 receptor by TGF*β*1, TGF*β*1-induced receptor kinase activation rapidly phosphorylates Smads proteins and initiates formation of functional oligomeric complexes. The resultant oligomeric complex translocates to the nucleus to regulate target gene expression. Briefly, the type I TGF*β*1 receptor kinase phosphorylates serine residues at the C-terminal SSXS motif in the MH2 domain of Smad 3 (or Smad 2) [24]. Phosphorylated Smad 3 (or Smad 2) forms an oligomeric complex with Smad 4, which is crucial for the maximal transcription of diverse TGF*β*1-inducible target genes [25, 26]. The oligomeric complexes of Smad 3 (or Smad 2) and Smad 4 recognize DNA binding element tetranucleotide (CAGA) or GC-rich sequences, and several copies of which are present in the promoter regions of many TGF*β*1-responsive genes such as plasminogen activator inhibitor-1 (PAI-1), *α*2(I) procollagen, and type VII collagen [25, 27]. It is well known that the protein products encoded from these genes promote the accumulation of extracellular matrix and that abnormal accumulation of the proteins may lead to fibrosis, which represents a form of the epithelial to mesenchymal transition (EMT).

Moreover, TGF*β*1-activated kinase-1, a member of MAPK kinase kinase family, activates its MAP kinase pathways [28, 29]. It is accepted that TGF*β*1-activated ERK pathway synergistically enhances Smad signaling of the TGF*β*1 receptor due to the positive cross talk between the ERK and Smad pathways [22, 30]. Serine phosphorylation of Smad 3/2, but not phosphorylation of the C-terminal motif, was decreased by MEK-ERK inhibitors [31]. Smad 3/2 are differentially activated by TGF*β*1 in hepatic stellate cells as a result of the differential phosphorylations of the Smads. Smad 3 plays a key role in TGF*β* signaling, which is strengthened by the observation that the loss of Smad 3 interfered with TGF*β*1-mediated induction of target genes [32, 33]. In addition, activation of CCAAT/enhancer binding protein (C/EBP) *β* is also involved in the inhibition of TGF*β*1 expression [34].

During the process of carcinogenesis, TGF*β* action can be either tumor suppressive or tumor promoting, depending on the stage of tumor development [35–37]. In an experimental cell model, TGF*β* could induce cell growth arrest and promote apoptosis of carcinoma cells [1]. The antiproliferative action of TGF*β* in epithelial cells, for example, is essentially attributed to the cell cycle arrest and the apoptosis concomitantly induced. It is well known that cell cycle arrest induced by TGF*β* occurs at G1 phase through enhancing transcription of cyclin-dependent kinase inhibitors, p21^Cip1/WAF^ and p15^Ink4b^, while suppressing the induction of c-Myc, a progrowth transcription factor, and of Id_1–3_, the inhibitors of differentiation [38–43]. In a model of gastric adenocarinoma, TGF*β*-mediated apoptosis contributed to tumor suppression, which resulted from TGF*β*-induced caspase-8 activation [44]. Moreover, it has been shown that TGF*β* reduced the expression of antiapoptotic Bcl-2 family members in prostate cancer cells [45].

By contrast, TGF*β* may also lead to tumor cell proliferation as a consequence of EMT process [46–48], which is a cellular phenomenon characterized by a loss of polarized epithelial phenotype with transition to a mesenchymal or more migratory phenotype. Studies have shown that diverse signaling pathways are involved in the TGF*β*-dependent EMT process. Initiation of EMT by TGF*β* receptor activation is mediated by either Smad-dependent or Smad-independent pathway [1, 49, 50]. Downstream of the TGF*β* receptor activation, the Smads activated by the TGF*β* receptor kinase promote transcription of the genes, which eventually play crucial roles in the process of EMT. The responsible transcription factors primarily include Snail, Slug, and LEF-1 [1]. In addition, TGF*β* also activates the non-Smad pathways, which include Ras, phosphatidylinositol 3-kinase (PI3K), and Par 6. These molecules regulate the expression of Snail and the activities of glycogen synthase kinase 3*β* (GSK3*β*) and RhoA, respectively [51, 52], thereby enhancing the process of EMT. It is now accepted that the EMT phenomenon of primary cancer cells promoted by the action of TGF*β* may increase cancer metastasis.

TGF*β* acts on tumor cells directly, playing a role in cancer cell migration and invasion. Diverse TGF*β*-mediated signaling pathways are responsible for this process. In glioblastoma cells, siRNA knockdowns of TGF*β*1 and TGF*β*2 resulted in the inhibition of cell motility or invasiveness [53]. As a same token, TGF*β* released from tumor tissues might facilitate glioma cell migration and invasion via an autocrine signaling [54]. Several lines of evidence also support the concept that TGF*β*-induced Smad signaling is responsible for the invasiveness of cancer cells [55–58]. This is explained in part by the TGF*β*-dependent induction of matrix metalloproteases, which are known to be responsible for cell migration and invasion [55, 59–62]. Activation of ERK and JNK by TGF*β* and their association with focal complexes may also contribute to cell migration, as shown in the case of breast carcinoma [63]. Moreover, it has been shown that the activation of p38 MAPK pathway by TGF*β* facilitated invasion of head and neck squamous epithelial cells [61].

In addition to the double-edged effects of TGF*β* on cancer cells, TGF*β* may alter cancer growth by suppressing the growth of multiple immune cells, which compromises the overall immune functions. Studies have shown that the proliferation and activity of T cells are suppressed by the TGF*β* blockade of IL-2 production and expression of T cell effector molecules [64–68]. Also, TGF*β* attenuates the activity of natural killer (NK) cells by inhibiting NK production of interferon-*γ* (IFN-*γ*) [69, 70]. Another study showed that TGF*β* inhibited the antigen presentation function of dendritic cells through suppressing the expression of MHC class II and costimulatory molecules [71]. All of these results support the alterations by TGF*β* in immune functions, which would impair immune surveillance or attack against cancer cells.

**Figure 1:**
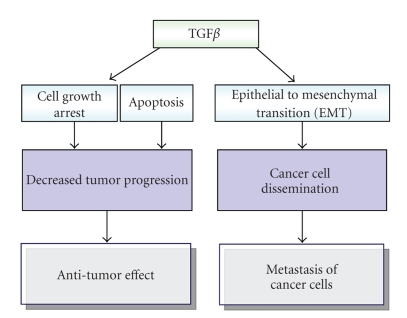
A scheme showing the opposing effects of TGF*β* on tumor growth and metastasis.

In summary, action of TGF*β*1 on cancer cells switches from tumor suppression to tumor promotion, depending on the stage of tumor progression. For instance, during the early phase of breast tumorigenesis, the TGF*β* signal inhibits primary tumor growth via cell growth arresting and promoting apoptosis. However, at later stage, cancer cells acquire a capacity to escape from the tumor suppressive effects of TGF*β*1 via induction of EMT. Interestingly, the aforementioned conflicting functions of TGF*β* might go through the same TGF*β* receptor complex and the associated signaling pathways involving Smad transcription factors [1]. Probably, there should be certain stage-dependent modifications in cellular signaling system including changes in receptor function and downstream Smad signaling cascades. Taken together, it is concluded that TGF*β* may not only induce growth arrest of cancer cells, but also increase cancer dissemination [1], supporting the concept that the cytokine serves a dual function in tumor development and progression (Figure 1).

## 3. PPAR*γ* AND CANCER BIOLOGY

PPAR*γ* has been extensively studied as an anticancer target in preclinical and clinical settings [72]. The anticancer effects appeared to be cancer cell-specific. A knock-out or loss of function mutation in PPAR*γ* can be an important risk factor for the incidence of cancer [73–75]. In this sense, PPAR*γ* has been considered as a novel target for designing new anticancer drugs for chemotherapy. This is further supported by the finding that PPAR*γ* activators exert a potent tumor-suppressing activity against various human cancer cells [76–78]. As a matter of fact, PPAR*γ* activators such as troglitazone and ciglitazone exert antiproliferative activities in epithelial cancer cell lines or animal models, which presumably results from the activation of PPAR*γ* receptor and the PPAR*γ* receptor-dependent pathways [76, 79–83]. Nevertheless, other anticancer pathways have also been recognized in association with PPAR*γ*, which might be PPAR*γ* receptor-independent [84, 85]. Multiple PPAR*γ*-independent anticancer targets of PPAR*γ* agonists have been suggested in several cancer cell types. The mechanisms may comprise a variety of pathways such as the blockade of G1-S phase transition by inhibiting translation initiation [86], activation of JNK-dependent cell death pathway [87], induction of the early growth response-1 (Egr-1) gene [88], inhibition of Bcl-xL and Bcl-2 function [85], counteracting TGF*β* release by tumor cells [54], and induction of cyclin-dependent kinase inhibitor p21^WAF1/CIP1^ [89]. However, the precise antiproliferative mechanisms of the PPAR*γ* agonists remain to be further studied. On the contrary, there are also other reports available on the opposite effects showing that PPAR*γ* signaling promoted carcinogenesis [90, 91].

It should be noted that the antitumor effects of PPAR*γ* may be explained at least in two different ways. One mechanism involves cell growth regulation [4], which should be further clarified, whereas the other mechanism includes cancer chemopreventive effects mediated by the induction of antioxidant enzymes [92]. It is well recognized that PPAR*γ* affects cell survival, growth, and differentiation by acting on the peroxisomal proliferator-response element (PPRE), thereby modulating an expression of a group of genes controlling cell growth and differentiation pathways [93, 94]. The PPAR*γ* homodimer and PPAR*γ*-retinoic acid X receptor (RXR) *α* heterodimer have the specificities of DNA-binding with preferential binding of the latter to DR1, which is a PPRE DNA binding site. SRC-1 is a coactivator of PPAR*γ* [95]. Binding of the ligand-activated PPAR*γ*-RXR*α* heterodimer to its DNA binding sites stimulates the interaction between PPAR*γ*-RXR*α* and p160/SRC-1 [95].

A number of studies support the concept that cancer chemoprevention is accomplished by the induction of antioxidant enzymes. The results from our laboratories indicated that oltipraz and flavonoids as potential cancer chemopreventive agents activate C/EBP*β* in the antioxidant genes such as *glutathione S-transferase (GST) A2* [96, 97]. In addition, treatments of cells with PPAR*γ* activators induced the nuclear translocation of NF-E2-related factor 2 (Nrf2) and C/EBP*β*, and activating Nrf2 and C/EBP*β* bindings to the antioxidant response element (ARE) and C/EBP response elements, respectively [92]. Moreover, the Nrf2 and C/EBP*β* genes contain PPRE sites, which account for the induction of the target antioxidant proteins by PPAR*γ* activators. Both the ARE and the C/EBP binding site have crucial roles in transactivating the GSTA2 gene by PPAR*γ* and RXR ligands [92]. Therefore, Nrf2 and/or C/EBP*β* inductions(s) via the PPAR*γ* and RXR*α* heterodimer binding to the PPREs in the promoter regions of the target genes contribute(s) to the antioxidant capacity of cells (e.g., GSTA2).

A result of our previous study indicated that specific mutations of these nuclear binding sites in the GSTA2 promoter, which are present as a three-PPRE cluster, caused the complete loss of its responsiveness to PPAR*γ* activators [92]. All of the putative PPRE sites comprising DR1 were functionally active. Therefore, the binding of the activating PPAR*γ*-RXR heterodimer to all of the PPRE sites appeared to be crucial for the inducible gene activation, showing that the PPAR binding site cluster is the functionally active PPRE-responsive enhancer module (PPREM) [92]. This study on the regulation of gene expression by the PPAR*γ*-RXR heterodimer at the promoter containing multiple DR1 elements brought additional insight into the transcriptional control mechanism of the antioxidant enzymes. The identified molecular mechanism would shed light on the contribution of cell viability and cancer chemoprevention as a consequence of the induction of antioxidant targets genes by PPAR*γ* activators.

## 4. TGF*β* REGULATION BY PPAR*γ*-RXR AND CELL SIGNALING

Activation of the PPAR*γ*-RXR heterodimer represses the TGF*β*1 gene through dephosphorylation of a transcription factor called zinc finger transcription factor-9 (Zf9), which has been shown to be induced by phosphatase and tensin homolog deleted on chromosome (PTEN)-mediated p70 ribosomal S6 kinase-1 (S6K1) inhibition [18]. Because RXRs are modular proteins with a highly conserved central DNA binding domain and a less conserved ligand binding domain [98], activation of the PPAR*γ* and RXR heterodimer contributes to the gene regulation. The role of PPAR*γ* in repression of the TGF*β*1 gene was further evidenced by the effects of thiazolidinediones, and also by the reversal of TGF*β*1 repression by the dominant negative mutants, supporting to the novel aspect that PPAR*γ* activation contributes to TGF*β*1 gene repression and that RXR*α* is necessary for the full responsiveness in the gene repression. In fact, the inhibition of TGF*β*1 gene by the PPAR*γ* and RXR heterodimer might account for either tumor suppression or tumor promotion [18]. Also, as an effort to identify the molecular basis of TGF*β*1 repression by PPAR*γ* activators, the effects of PPAR*γ* and RXR activation on the TGF*β*1 gene transactivation, that is regulated by the proximal DNA response elements, have been examined [18]. The potential regulatory sites responsible for the TGF*β*1 gene expression have been explored by using the luciferase reporter gene assays, which identified the putative PPREs located at the multiple sites upstream from −453 bp of the promoter region [18]. Promoter deletion analyses indicate that neither the putative PPREs nor the activator protein-1 (AP-1) binding sites are directly regulated by PPAR*γ* activators forthe gene repression.

**Figure 2:**
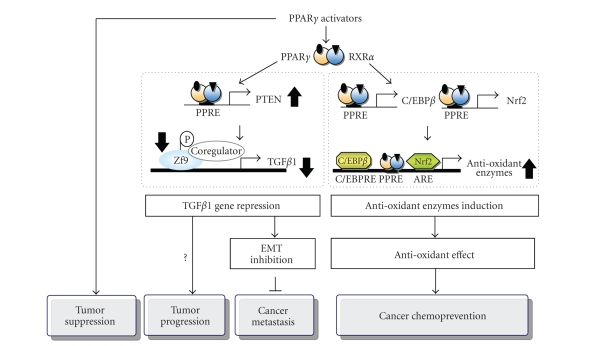
A schematic presentation of the multiple pathways regulated by PPAR*γ* for tumor suppression, progression, inhibition of metastasis, and cancer chemoprevention.

S6K1, a ubiquitous serine/threonine kinase, controls the translational efficiency by phosphorylating ribosomal S6 protein [99]. S6K1 functions as a multifunctional kinase for the phosphorylation of ribosomal S6 protein [99], CREM [100], BAD [101], and the eukaryotic elongation factor 2 kinase [102]. Rapamycin, a well-known mammalian target of rapamycin (mTOR) inhibitor, inhibited liver fibrosis and TGF*β*1 expression in rats bile duct-ligated or challenged with toxicants [103, 104], with a concomitant decrease in S6K1 activity. It is well recognized that rapamycin inhibits S6K1 activity via mTOR inhibition [105]. Yet, other pharmacological agents that modulate S6K1 activity have not been reported. The mechanism of PPAR*γ*-RXR heterodimer-mediated repression of the TGF*β*1 gene has been elucidated in terms of the modulation of S6K1 activity (Figure 2).

The PI3K-mTOR pathway regulates S6K1 for the regulation of transcription factors involved in the TGF*β*1 gene transactivation. A study identified the inhibition of S6K1 activity by the PPAR*γ*-RXR, which contributes to TGF*β*1 gene repression [18]. Another signaling molecule, PTEN, antagonizes the PI3-kinase-mTOR-S6K1-mediated signaling cascade [106, 107]. Thus, it has been elucidated that PPAR*γ* activators upregulate PTEN, which leads to the S6K1 inhibition, consequently causing TGF*β*1 repression [18].

## 5. TRANSCRIPTION FACTORS RESPONSIBLE FOR TGF*β* REPRESSION BY PPAR*γ*-RXR

**Figure 3:**
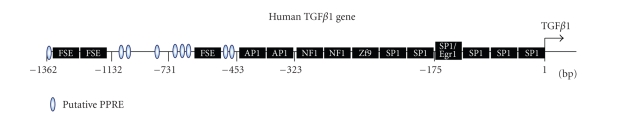
The human TGF*β*1 promoter region.

In the promoter region of the TGF*β*1 gene (Figure 3), the putative binding sites for PPAR*γ*-RXR seemed to be neither active nor responsible for the gene repression by the activated PPAR*γ* and RXR heterodimer. It has been claimed that the effects of PPAR*γ* or retinoid ligands on TGF*β*1 gene expression might be mediated in part by AP-1 inhibition [108, 109]. Nevertheless, such a result that deletion of the DNA region containing both AP-1 sites still had the capability to repress the gene by PPAR*γ* activator suggests that the AP-1 binding sites might not be a major regulatory target in the TGF*β*1 gene repression. Rather, the target molecule altered by PPAR*γ*-RXR*α*-activated cell signal may be involved in the interaction with the protein recruited on the AP-1 DNA complex. It appeared that the TGF*β*1 gene repression may have not resulted from the direct inhibition of AP-1, but other mechanistic basis [18].

Another study showed that the mechanism associated with the inhibition of TGF*β*1 by PPAR*γ* activators involves the regulation of c-Fos [108]. In the study, thiazolidinediones inhibit high-glucose-induced TGF*β*1 promoter activity. A suggested mechanism was raised based on the observation that treatments of thiazolidinediones reduced high-glucose-induced, activated PKC and c-Fos-mediated TGF*β*1 gene expression in mesangial cells [108].

Zf9 as an immediate early gene reduces cell proliferation with the induction of p21^cip1^ and the enhancement of c-Jun degradation [110, 111], thus functioning as a potential tumor suppressor gene. The transcription factors that interact with the known DNA binding sites on the region downstream within the −323 bp of the TGF*β*1 gene include Zf9, NF1, and SP1. It is noteworthy that Zf9 activation induces TGF*β*1 during the activation of hepatic stellate cells [112]. Also, Zf9 regulates TGF*β* receptors and collagen *α*1(I), promoting accumulation of extracellular matrix [113]. Studies have shown that Zf9 phosphorylation enhances its nuclear localization and transcriptional activity [111]. Zf9 as a transcription factor plays a crucial role for the induction of TGF*β*1 [113]. Thus, phosphorylation status of Zf9 may contribute to the promotion of its target gene expression [114]. Identification of the partners of Zf9 or phosphorylated Zf9 for the TGF*β*1 gene regulation and their molecular interactions would be interesting to pursue. The constitutive Zf9 phosphorylation by S6K1 strengthened the important role of S6K1 as a multifunctional kinase for the transcription factor regulation of target genes [100–102].

The TGF*β*1 gene contains the DNA response element interacting with Zf9 [16] that regulates multiple genes involved in tissue differentiation. Activation of Zf9 includes its phosphorylation at serine (or tyrosine) residues [114]. Thus, phosphorylation of Zf9 leads to transcription of its target genes [111, 114]. Although the kinase catalyzing Zf9 phosphorylation has not been completely identified, the inhibition of Zf9 phosphorylation by rapamycin that inhibits S6K1 activity via mTOR inhibition supports the role of S6K1 in Zf9 phosphorylation [18]. More importantly, the role of S6K1 in regulating TGF*β*1 gene and the associated molecular mechanistic basis have been clarified in terms of Zf9 dephosphorylation [18]. In view of the previous observations that Zf9 is crucial as a transcription factor for TGF*β*1 induction in hepatic stellate cells [113] and that a phosphorylated form of Zf9 plays a role in the transactivation of the target gene promoter [114], the potential ability of PPAR*γ* activators to inhibit serine phosphorylation of the transcription factor has also been investigated. Thus, it has been demonstrated that the inhibition of the TGF*β*1 gene by the activation of PPAR*γ*-RXR includes Zf9 dephosphorylation [18]. Therefore, TGF*β*1 gene repression by PPAR*γ* activators appears to be related with dephosphorylation of Zf9, supporting the conclusion that the PPAR*γ*-RXR heterodimer causes TGF*β*1 repression via S6K1 inhibition, and that the inhibition of S6K1 activity provides a central mechanism, by which PPAR*γ*-RXR regulates Zf9-dependent TGF*β*1 gene expression (Figure 2).

Moreover, it has been shown that PPAR*γ* activation induces PTEN, which serves as a PI(3,4,5)P_3_ lipid phosphatase and antagonizes PI3-kinase-mediated cell signaling [106]. Functional PPREs located in the PTEN promoter have been recognized [115]. The induction of PTEN by PPAR*γ* activators may result in TGF*β*1 gene repression following S6K1 inhibition. Furthermore, PPAR*γ* activators inhibited phosphorylations of Akt, ERK1/2, p90 ribosomal S6 kinase-1 (RSK1), and mTOR, downstream of PTEN, indicating that PTEN induction by PPAR*γ* activators leads to S6K1 inhibition via the pathways of ERK1/2-RSK1 as well as Akt-mTOR. In conclusion, the result showing that PPAR*γ* activation upregulates PTEN, which has also been implicated in tumor-inhibitory or anti-inflammatory actions of PPAR*γ* [106, 115], gives credence to the concept that PPAR*γ* activators induce PTEN during S6K1 inhibition, and consequently causes TGF*β*1 repression. Therefore, the inhibition of tumor proliferation by PPAR*γ* activators may be explained in part by PPAR*γ*-dependent TGF*β*1 repression (Figure 2), supporting the concept that the PPAR*γ* activators may be applied for controlling TGF*β*1-induced cancer metastasis and fibrosis.
